# Light Chain Deposition Disease Diagnosed Using Computed Tomography-Guided Kidney Biopsy

**DOI:** 10.7759/cureus.15102

**Published:** 2021-05-18

**Authors:** Yoshinosuke Shimamura, Yayoi Ogawa, Hideki Takizawa, Toshiaki Hayashi, Yasuo Sakurai

**Affiliations:** 1 Department of Nephrology, Teine Keijinkai Medical Center, Sapporo, JPN; 2 Department of Renal Pathology, Hokkaido Renal Pathology Center, Sapporo, JPN; 3 Department of Hematology, Teine Keijinkai Medical Center, Sapporo, JPN; 4 Department of Radiology, Teine Keijinkai Medical Center, Sapporo, JPN

**Keywords:** bortezomib, computed tomography, kidney biopsy, daratumumab, light chain deposition disease

## Abstract

Light chain deposition disease (LCDD) is characterized by the deposition of monoclonal immunoglobulin light chains in the kidney, which can cause end-stage kidney disease if not treated. While kidney biopsy is required for definitive diagnosis, choosing an appropriate biopsy method may be problematic when examining patients with atrophic kidneys. A 66-year-old Japanese man was referred to our institution with a three-month history of leg edema. Clinical investigations revealed proteinuria levels of 7.5 g/day. CT-guided percutaneous kidney biopsy was selected as the biopsy method because atrophic kidneys were poorly visualized on ultrasonography. Kidney biopsy revealed nodular glomerulosclerosis, exclusive deposition of the κ chain, and powdery electron-dense deposits, all of which were indicative of LCDD. Bence-Jones protein was detected in the urine. The patient also had an abnormal serum-free light chain ratio. Bone marrow biopsy revealed multiple myeloma; therefore, the patient was diagnosed to have LCDD with multiple myeloma. The patient was treated with daratumumab, bortezomib, cyclophosphamide, and dexamethasone. After a one-year follow-up, the patient had hematological and renal responses without any treatment-related adverse effects. Our case demonstrates the effectiveness of daratumumab as a treatment for LCDD with nephrotic-range proteinuria. Additionally, we suggest that CT-guided kidney biopsy should be considered as a diagnostic test in patients with kidney atrophy when making a definitive diagnosis.

## Introduction

Light chain deposition disease (LCDD) is a type of monoclonal immunoglobulin deposition disease characterized by the deposition of non-amyloid monoclonal immunoglobulin light chains in various organs, including the kidney, heart, and liver [1-3]. The main renal manifestations include proteinuria, hematuria, and kidney failure [1], and LCDD may progress to end-stage kidney disease, which requires renal replacement therapy in the absence of early diagnosis and treatment [4]. Autologous hematopoietic cell transplantation is indicated for patients with LCDD [5, 6], while treatment with bortezomib, cyclophosphamide, and dexamethasone is indicated for transplant-ineligible patients [4, 7]. Additionally, the effectiveness of daratumumab, an anti-CD38 monoclonal antibody, has been previously reported [8-11].

A kidney biopsy is required for the definitive diagnosis of LCDD. Although ultrasound-guided percutaneous kidney biopsy is the standard method, a CT-guided approach is a possible alternative when the kidneys are difficult to visualize, as with small echogenic kidneys [12-14]. Herein, we report the case of a patient with LCDD who was diagnosed using CT-guided percutaneous kidney biopsy and successfully treated with daratumumab, bortezomib, cyclophosphamide, and dexamethasone. This report highlights the importance of early and aggressive treatment in patients with LCDD to achieve a hematologic response and improve renal prognosis.

## Case presentation

A 66-year-old Japanese man was referred to our medical center with leg edema. He had hypertension and dyslipidemia. His vital signs were unremarkable. Notable examination findings included moderate pitting edema in the lower extremities. Neurological and musculoskeletal examination findings were unremarkable. Laboratory test results (Table 1) revealed a normal red blood cell count (440 × 104/μL [normal: 427-520 × 104/μL]), normal white blood cell count (4,400/μL [normal: 3,040-8,540/μL]), and normal platelet count (20.7 × 109/L [normal: 15.036.1 × 109/L]). Serum creatinine was 1.59 mg/dL, estimated glomerular filtration rate was 45 mL/min/1.73 m2, and serum albumin was 3.4 g/dL. Immunological analysis showed decreased immunoglobulins (IgG 291 mg/dL; IgA 16 mg/dL; IgM 21 mg/dL), and normal complement levels. The patient tested negative for antinuclear, double-stranded DNA, anti-Smith, antineutrophil cytoplasmic, and anti-glomerular basement membrane antibodies. The patient’s cryoglobulin test, as well as tests for hepatitis A, B, C, and HIV, was negative. Urinalysis showed profound proteinuria (7.5 g/day [normal: <0.02 g/day]) with mild hematuria (10-19 per high-power field [normal: <1 per high-power field]).

**Table 1:** Laboratory findings at the initial evaluation.

Laboratory data	At the initial evaluation	Reference ranges
Red blood cell count (× 10^4^/μL)	440	427–520
White blood cell count (/μL)	4400	3040–8540
Platelet count (× 10^9^/L)	20.7	15.0–36.1
Blood urea nitrogen level (mg/dL)	35.9	8–20
Serum creatinine level (mg/dL)	1.59	0.5–0.8
Estimated glomerular filtration rate (mL/min/1.73 m^2^)	45	≥60
Total protein level (g/dL)	5.1	6.6–8.0
Serum albumin level (g/dL)	3.4	4.1–5.0
Serum calcium level (mg/dL)	8.7	8.5–10.2
Bilirubin level (mg/dL)	0.6	0.3–1.2
Alanine transaminase level (U/L)	20	5–45
Aspartate aminotransferase level (U/L)	16	5–45
Alkaline phosphatase level (U/L)	209	104–398
Lactate dehydrogenase level (U/L)	122	120–145
Serum IgG (mg/dL)	291	870–1700
Serum IgA (mg/dL)	16	90–140
Serum IgM (mg/dL)	21	35–220
C3 (mg/dL)	92	65–135
C4 (mg/dL)	35	13–35
CH50 (IU/mL)	45	28–53
Urine protein (g/day)	7.5	<0.02
Microscopic hematuria (/high-power field)	10–19	<1
Free light chain ratio	289	0.248–1.804
Serum N-terminal pro-B-type natriuretic peptide (pg/mL)	54.2	0–125
Troponin I level (pg/mL)	2.9	0–26

**Figure 1:**
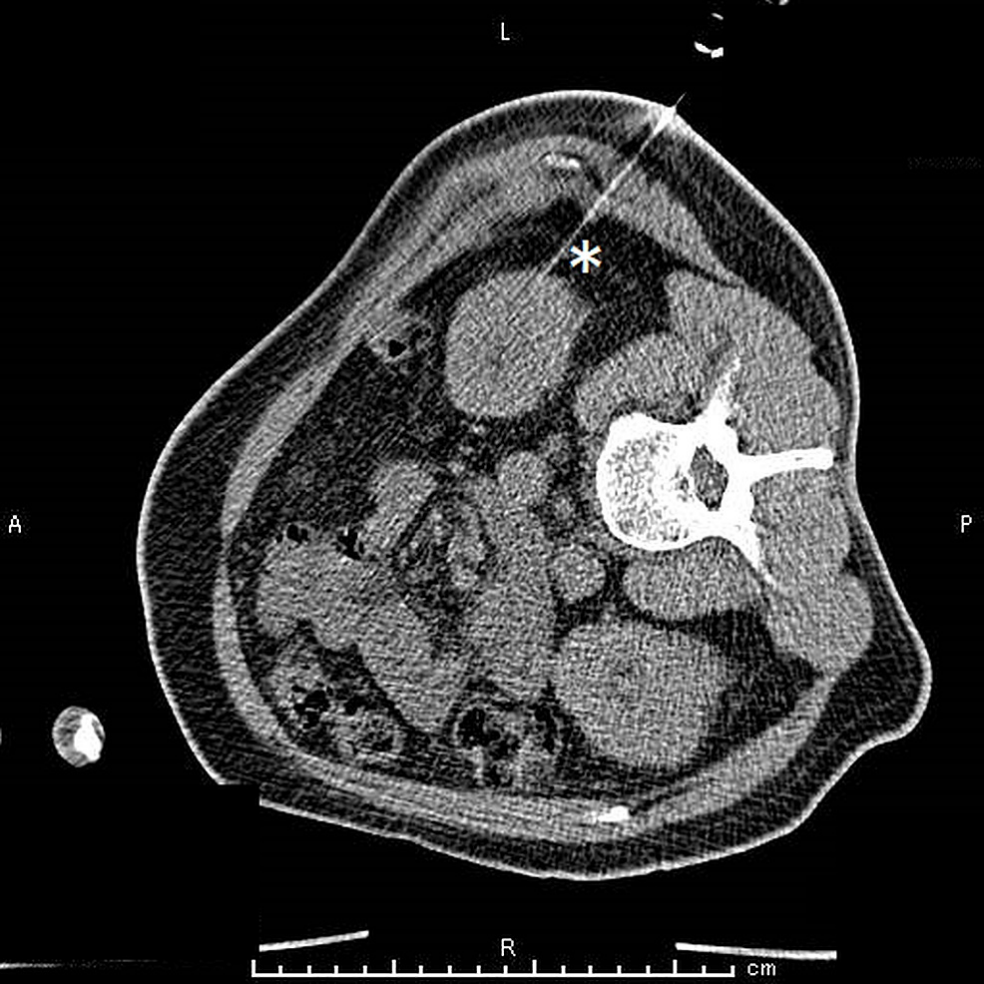
CT-guided kidney biopsy performed using a 17-gauge needle (white asterisk).

Since kidney atrophy (7 cm in length; 3.2 cm in width) was observed on ultrasonography, we performed a CT-guided percutaneous kidney biopsy (Figure 1).

**Figure 2:**
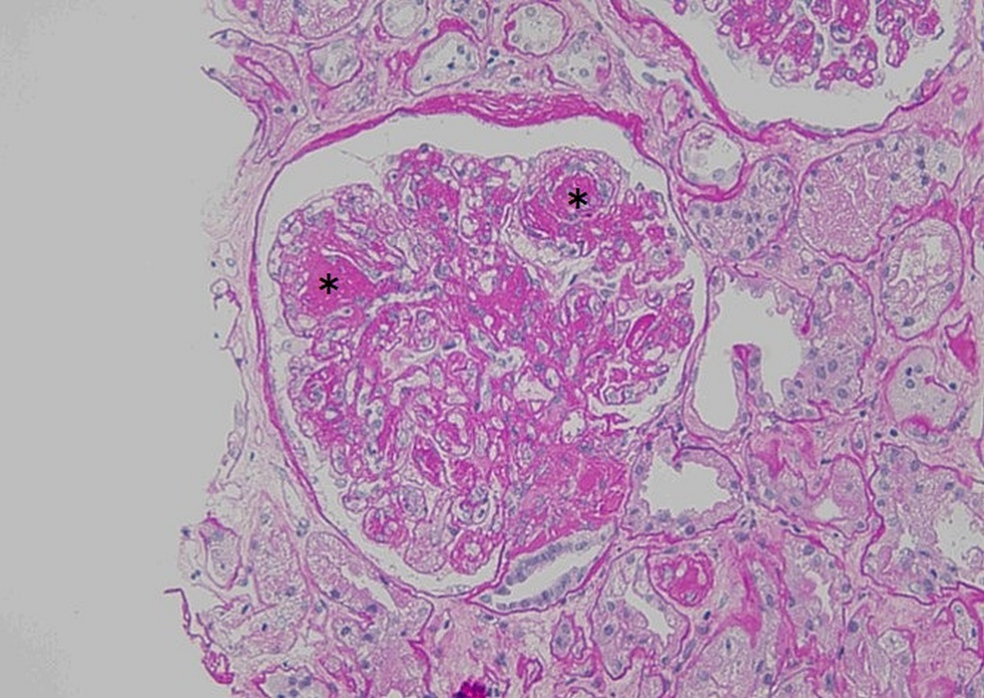
Light microscopy of the kidney showing nodular glomerulosclerosis (black asterisk) (periodic acid–Schiff stain, ×400).

**Figure 3:**
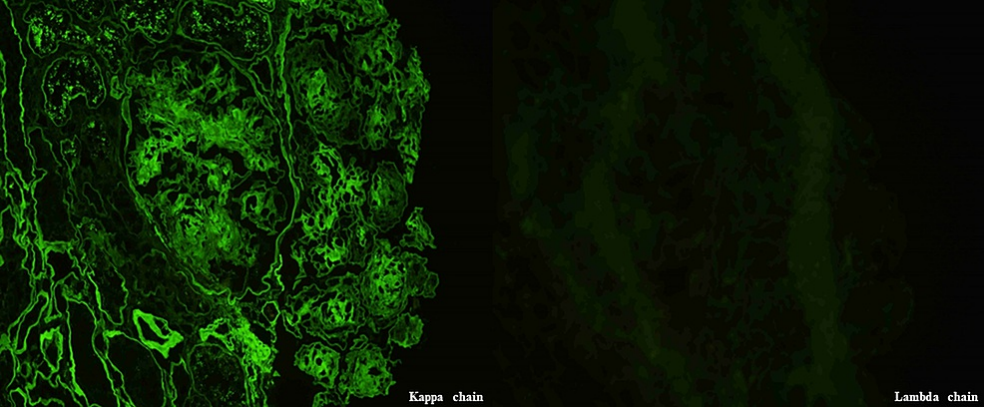
Immunofluorescence microscopy of the kidney showing an exclusive deposition of κ chain (left panel, κ chain immunofluorescence; right panel, λ chain immunofluorescence, ×400).

**Figure 4:**
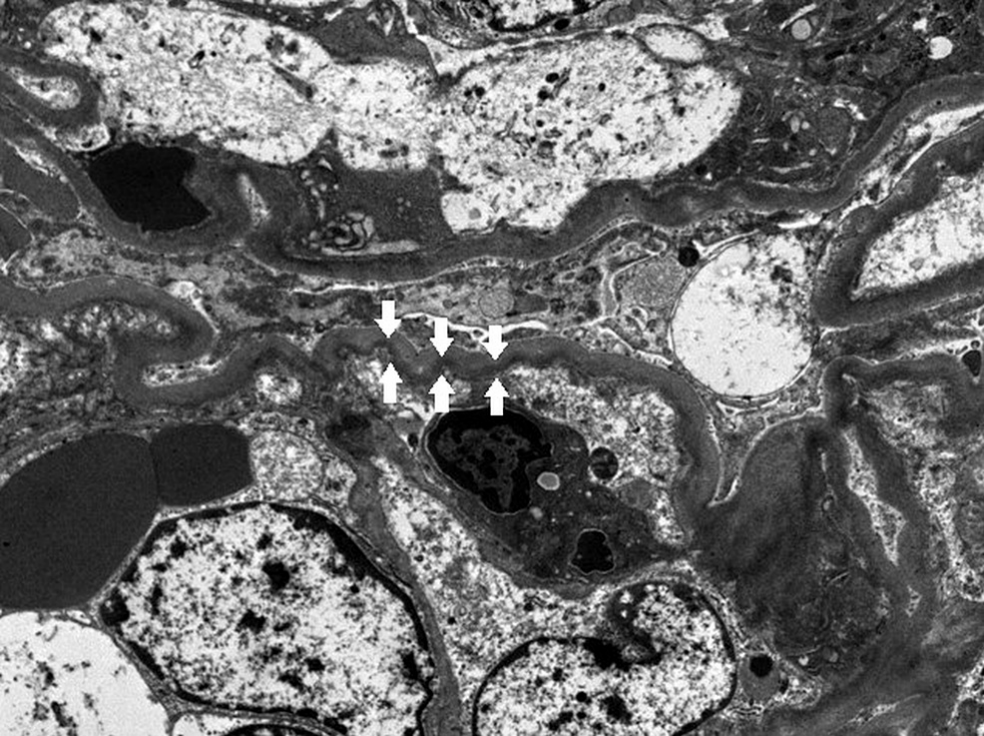
Electron microscopy of the kidney showing powdery continuous electron-dense deposits in the subendothelial area (white arrows) (electron microscopy, ×1500).

Light microscopy revealed 24 glomeruli with nodular glomerulosclerosis accompanied by mild mesangial hypercellularity (Figure 2), and immunofluorescence microscopy revealed exclusive deposition of κ chain along the glomerular basement membrane (Figure 3). Congo red staining was negative. Electron microscopy revealed continuous powdery electron-dense deposits in the subendothelial area (Figure 4), which supported the diagnosis of LCDD.

Serum immunofixation revealed a monoclonal IgG κ band with a free light chain (FLC) ratio of 289 (κ chain, 3180 mg/dL: λ chain, 11 mg/dL). Bone marrow biopsy showed 19% plasma cell infiltration by CD138 immunostaining. Flow cytometry showed that 8% of monoclonal plasma cells were restricted to the κ chain and were positive for CD56 and negative for CD19. No cytogenetic abnormalities were detected using fluorescence in situ hybridization. The final diagnosis was LCDD caused by multiple myeloma. The patient’s serum N-terminal pro-B-type natriuretic peptide level was 54.2 pg/mL (normal: 0-125 pg/mL) and troponin I level was 2.9 pg/mL (normal: 0-26 pg/mL). Transthoracic echocardiography revealed that the patient’s interventricular septum thickness was 9.9 mm, suggesting a lack of cardiac involvement. A liver biopsy was not performed because the patient did not consent. The patient was deemed ineligible for autologous hematopoietic cell transplantation because of nephrotic-range proteinuria. Treatment with bortezomib, cyclophosphamide, and dexamethasone, followed by weekly daratumumab (16 mg/kg), was initiated. Acyclovir and trimethoprim-sulfamethoxazole were added for prophylaxis. After a one-year follow-up, proteinuria levels declined from 7.5 to 0.3 g/day, hematuria resolved, serum creatinine level decreased from 1.59 to 1.21 mg/dL, and the difference between involved FLC and uninvolved FLC (dFLC) decreased from 3169 to 200 mg/dL. At the time of reporting this study, the patient had been taking daratumumab, bortezomib, cyclophosphamide, and dexamethasone with a very good partial response. No treatment-related adverse effects, including infections, cytopenia, and peripheral neuropathy, have been observed to date. We performed a serum FLC assay and 24-hour urine protein measurement to monitor the treatment response during monthly outpatient visits. We also performed liver function tests and N-terminal pro-B-type natriuretic peptide measurements every three months to evaluate the emergence of cardiac and hepatic manifestations.

## Discussion

In the present case, we made two important observations. First, LCDD needs to be diagnosed and treated as early as possible to achieve favorable hematologic and renal responses. Our patient achieved a very good partial response to treatment with daratumumab, bortezomib, cyclophosphamide, and dexamethasone, resulting in improved kidney function and urine abnormalities. This could be attributed to the early recognition and diagnosis of LCDD. A previous observational study [2] had reported that hematologic responses to chemotherapy were associated with favorable renal outcomes in patients with LCDD. Another study [4] demonstrated that a baseline glomerular filtration rate <20 mL/min/1.73 m2 and renal response were independent prognostic factors for progression to dialysis. In addition, Cohen et al. [7] reported that the renal response rate was significantly higher in patients with stage 1-3 chronic kidney disease than in those with stage 4-5 chronic kidney disease among LCDD patients who received bortezomib-based treatment regimens. In this case, the patient’s serum creatinine levels and estimated glomerular filtration rate were 1.59 mg/dL and 45 mL/min/1.73 m2, respectively, when chemotherapy was initiated. Another explanation for the improvement in renal parameters could be the rapid hematologic response to chemotherapy. The standardized treatment of LCDD remains inconclusive, mainly because of disease rarity and the lack of randomized controlled trials; however, several retrospective studies have reported the efficacy of combination chemotherapy with bortezomib, cyclophosphamide, and dexamethasone [4] [7]. Additionally, daratumumab, a CD38 monoclonal antibody, is effective in patients with multiple myeloma and those with monoclonal gammopathy of renal significance (MGRS), including AL amyloidosis, proliferative glomerulonephritis, monoclonal immunoglobulin deposits, and LCDD [8-10,11,15,16]. For example, Milani et al. [10] reported the effectiveness of daratumumab in improving renal function in patients with LCDD. Similarly, our patient demonstrated a rapid decline in dFLC levels from 3169 to 354 mg/dL one month after initiating daratumumab-based therapy, possibly contributing to the improvement of renal parameters. 

Second, our findings indicate that CT-guided percutaneous kidney biopsy is a useful alternative to the ultrasonography-guided technique in patients with atrophic kidneys [13, 14]. Current imaging-guided percutaneous kidney biopsy techniques, including ultrasonography or CT, can be performed more safely and less invasively than open biopsy [12]. Ultrasonography-guided techniques have the advantages of real-time needle placement and no radiation risk but are not recommended when kidneys cannot be well-visualized [12]. In contrast, CT-guided techniques can visualize the kidney more clearly because of their better resolution and tissue contrast [13]. We decided to perform a CT-guided kidney biopsy, which enabled us to collect adequate tissue samples without any postprocedural complications such as perinephric bleeding [14].

## Conclusions

We report a case of LCDD that was diagnosed using CT-guided kidney biopsy and successfully treated with daratumumab-based chemotherapy. Our case illustrates the utility of CT-guided percutaneous kidney biopsy in cases where performing the standard ultrasonography-guided technique is difficult. We suggest that clinicians should attempt to make a definitive diagnosis of LCDD by performing CT-guided percutaneous kidney biopsy because correct diagnosis and early treatment are prerequisites for successful treatment.
